# A scoping review of the changing landscape of geriatric medicine in undergraduate medical education: curricula, topics and teaching methods

**DOI:** 10.1007/s41999-021-00595-0

**Published:** 2022-01-01

**Authors:** Tahir Masud, Giulia Ogliari, Eleanor Lunt, Adrian Blundell, Adam Lee Gordon, Regina Roller-Wirnsberger, Michael Vassallo, Daniela Mari, Marina Kotsani, Katrin Singler, Roman Romero-Ortuno, Alfonso J. Cruz-Jentoft, Andreas E. Stuck

**Affiliations:** 1 Department of Health Care for Older People (HCOP), Queen’s Medical Centre, Nottingham University Hospitals NHS Trust, Derby Road, Nottingham, NG7 2UH Nottinghamshire UK; 2 Department of Geriatric Medicine, Odense University Hospital, Odense, Denmark; 3 University of Nottingham, Nottingham, UK; 4 University Hospitals of Derby and Burton NHS Foundation Trust, Derby, UK; 5 Department of Internal Medicine, Medical University of Graz, Auenbruggerplatz 15, 8036 Graz, Austria; 6 University Hospitals Dorset, Royal Bournemouth Hospital, Castle Lane East, Bournemouth, BH7 7DW UK; 7 Laboratory of Geriatric and Oncologic Neuroendocrinology Research, IRCCS Istituto Auxologico Italiano, Milan, Italy; 8 Université de Lorraine, CHRU-Nancy, Pôle “Maladies du Vieillissement, Gérontologie et Soins Palliatifs”, 54000 Nancy, France; 9 Working Group on the Development of Geriatric Medicine in Greece of the Hellenic Society for the Study and Research of Aging, 15342 Athens, Greece; 10 Department of Geriatric Medicine, Klinikum Nürnberg, Paracelsus Medical University Nürnberg, Nürnberg, Germany; 11 Institute for Biomedicine of Ageing, Friedrich-Alexander University Erlangen-Nürnberg, Nürnberg, Germany; 12 Discipline of Medical Gerontology, School of Medicine, Trinity College Dublin, Dublin, Ireland; 13 Hospital Universitario Ramón y Cajal (IRYCIS), Madrid, Spain; 14 Department of Geriatrics, University of Bern, 3010 Bern, Switzerland

**Keywords:** Geriatric medicine, Geriatric psychiatry, Undergraduate medical education, Curriculum, Teaching methods

## Abstract

**Purpose:**

The world’s population is ageing. Therefore, every doctor should receive geriatric medicine training during their undergraduate education. This review aims to summarise recent developments in geriatric medicine that will potentially inform developments and updating of undergraduate medical curricula for geriatric content.

**Methods:**

We systematically searched the electronic databases Ovid Medline, Ovid Embase and Pubmed, from 1st January 2009 to 18th May 2021. We included studies related to (1) undergraduate medical students and (2) geriatric medicine or ageing or older adults and (3) curriculum or curriculum topics or learning objectives or competencies or teaching methods or students’ attitudes and (4) published in a scientific journal. No language restrictions were applied.

**Results:**

We identified 2503 records and assessed the full texts of 393 records for eligibility with 367 records included in the thematic analysis. Six major themes emerged: curriculum, topics, teaching methods, teaching settings, medical students’ skills and medical students’ attitudes. New curricula focussed on minimum Geriatrics Competencies, Geriatric Psychiatry and Comprehensive Geriatric Assessment; vertical integration of Geriatric Medicine into the curriculum has been advocated. Emerging or evolving topics included delirium, pharmacotherapeutics, healthy ageing and health promotion, and Telemedicine. Teaching methods emphasised interprofessional education, senior mentor programmes and intergenerational contact, student journaling and reflective writing, simulation, clinical placements and e-learning. Nursing homes featured among new teaching settings. Communication skills, empathy and professionalism were highlighted as essential skills for interacting with older adults.

**Conclusion:**

We recommend that future undergraduate medical curricula in Geriatric Medicine should take into account recent developments described in this paper. In addition to including newly emerged topics and advances in existing topics, different teaching settings and methods should also be considered. Employing vertical integration throughout the undergraduate course can usefully supplement learning achieved in a dedicated Geriatric Medicine undergraduate course. Interprofessional education can improve understanding of the roles of other professionals and improve team-working skills. A focus on improving communication skills and empathy should particularly enable better interaction with older patients. Embedding expected levels of Geriatric competencies should ensure that medical students have acquired the skills necessary to effectively treat older patients.

**Supplementary Information:**

The online version contains supplementary material available at 10.1007/s41999-021-00595-0.

## Key summary points

**Aim:**

This scoping review aims to summarise recent developments in Geriatric Medicine that will potentially inform the updating of undergraduate medical curricula for geriatric content.

**Findings:**

Based on the thematic analysis of 367 records out of 2503 identified records, we summarised findings across six major themes: curriculum; topics; teaching methods; teaching settings; medical students’ skills and medical students’ attitudes.

**Message:**

This review could inform future shaping of undergraduate medical curricula for geriatric content, through model curricula, expansion of geriatric topics and use of various teaching methods and settings.

**Supplementary Information:**

The online version contains supplementary material available at 10.1007/s41999-021-00595-0.

## Introduction

Most doctors will encounter older adults in their practice, but the majority of older adults will not encounter a geriatrician [1]. Worldwide, the number of trained geriatricians per capita varies widely [2] and in many countries the specialty of Geriatric Medicine is still in its infancy [1, 3, 4]. Due to the population ageing and the medical complexity of older adults, every doctor should receive basic Geriatric Medicine training during their undergraduate education, aimed at developing knowledge, skills and attitudes relevant to older people [1]. Even where the teaching of Geriatric Medicine exists, there is considerable variation in its content and delivery across countries [1, 3, 4]. Harmonisation of undergraduate medical curricula across countries may facilitate the implementation of evidence-based practice and the international mobility of doctors.

In 2009, a European Summit on Age-Related Disease made strong recommendations on introducing geriatric content in the teaching of all healthcare professionals [3]. In 2014, the European undergraduate curriculum in Geriatric Medicine was developed through a Delphi process facilitated by the European Union of Medical Specialists- Geriatric Medicine Section (UEMS-GMS) [5]. In preparation for this, a literature review of existing curricula was undertaken [6]. The final curriculum was published as a list of overarching learning outcomes and corresponding specific learning objectives; educational material was published to support its implementation [7]. Since then, there have been further developments in clinical practice, research and teaching in Geriatric Medicine. Accordingly, the UEMS-GMS board and the Education Specialist Interest Group (SIG) of the European Geriatric Medicine Society (EuGMS) have recommended that the curriculum is updated. Moreover, it was felt that the new curriculum should incorporate content on different teaching methods that can be employed to teach Geriatric Medicine to undergraduates, as this was lacking in the previous version of the curriculum.

The aim of this scoping review was to summarise recent developments in undergraduate Geriatric Medicine to inform the next iteration of the European undergraduate medical curriculum in Geriatric Medicine. The specific objectives were to review: (1) any recent Geriatric Medicine undergraduate curricula; (2) new curricular topics or new developments in existing topics; (3) teaching methods and teaching settings in Geriatric Medicine; (4) medical students’ skills and attitudes in relation to Geriatric Medicine.

## Methods

### Search strategies

On 18th May 2021, we systematically searched the electronic databases Ovid Medline, Ovid Embase and Pubmed, using search algorithms developed with a clinical librarian as detailed in Supplementary Tables 1, 2 and 3. Our search terms combined three domains: (1) undergraduate medical students (or equivalent terms) AND (2) Geriatric Medicine or Ageing or Older People or Gerontology (or equivalent terms) AND (3) Curriculum or Education or Learning or Training or Teaching or Competencies or Objectives (or equivalent terms).

All records published online from 1st January 2009 to 18th May 2021 were included—2009 was the publication year of the previously mentioned literature review [6]. No language restrictions were applied and a translator was involved where necessary.

Through the UEMS-GMS network, we contacted experts in Geriatric Medicine, in Europe, Canada and Australia, to enquire about the existence of a national medical undergraduate curriculum in their countries.

### Selection criteria

Studies that met the following criteria were included: (1) related to undergraduate medical students; (2) related to Geriatric Medicine or ageing or older adults; (3) related to curriculum or curriculum topics or learning objectives or competencies or teaching methods or teaching settings or students’ attitudes towards older adults or Geriatric Medicine and (4) published in a scientific journal. We included studies on both medical and other healthcare students if they met the other criteria.

We excluded studies that: (1) did not specifically relate to undergraduate medical students but to other healthcare students; (2) did not specifically relate to Geriatric Medicine or ageing or older adults; (3) were related only to postgraduate medical education. We excluded these types of publication: (1) conference abstracts; (2) PhD theses and (3) medical students’ dissertations. We further excluded duplicates and translations of pre-existing curricula.

### Study selection

Three researchers (GO, EL, TM) independently screened titles and abstracts. Each record was screened for inclusion, based on title or abstract, by a pair of researchers working independently (GO and EL, GO and TM, TM and EL). Any disagreement was resolved through discussion between the two researchers or, if disagreement still persisted, through involvement of the third researcher.

### Data extraction and synthesis: theme-coding

Four researchers (GO, EL, TM, AB) coded each record, by identifying and generating themes or subthemes in it, based on the full-text. Over 42 themes emerged and were grouped into six major themes. Due to the heterogeneity and large numbers of papers, we presented our findings in a narrative way, by identifying themes and selecting a few papers per theme. We preferentially selected papers that proposed model curricula or novel curricular topics, or which were systematic reviews of teaching methods or surveys.

## Results

**Fig. 1:**
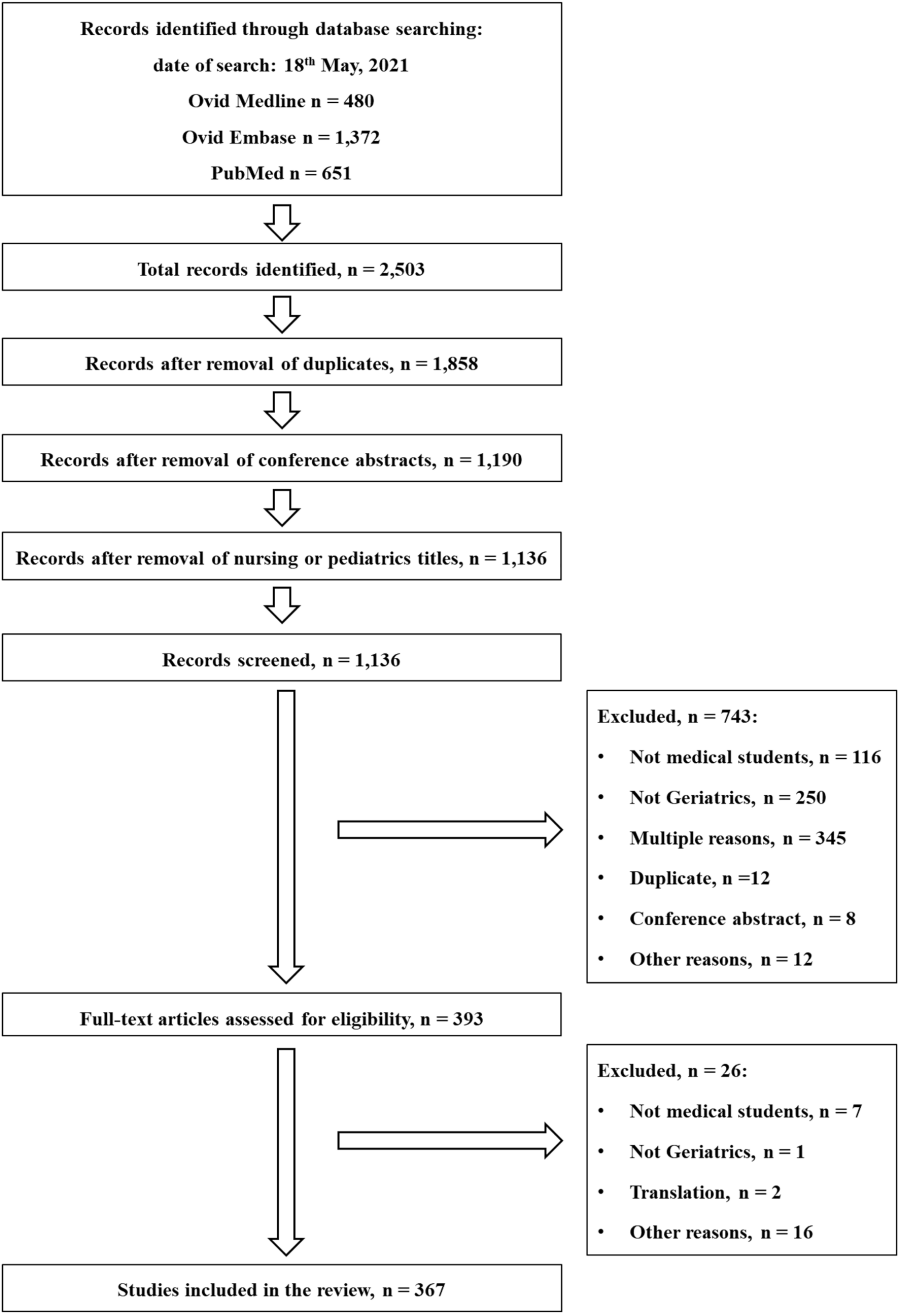
Flow-chart of studies selection

A total of 2503 records were identified from the electronic databases. After the removal of duplicates, conference abstracts and nursing or paediatric titles by the librarian, 1136 records remained. Following the screening of titles and/or abstracts, we excluded 743 records for the reasons shown in Fig. 1. The full texts of the remaining 393 records were assessed for eligibility and further 26 records were excluded. Finally, 367 records were included in the thematic analysis (Supplementary Excel file). Figure 1 details the search and study selection.

Of the 367 records, most were in English (*n* = 350), five in Spanish, four in Dutch, three in German, three in Japanese, one in French and one in Turkish. Records from high and low-middle income countries—and from all continents—were included.

Experts of the UEMS-GMS confirmed that a few countries have national recommendations on undergraduate medical education (e.g. France, UK, Germany, Switzerland and the Netherlands).

**Fig. 2:**
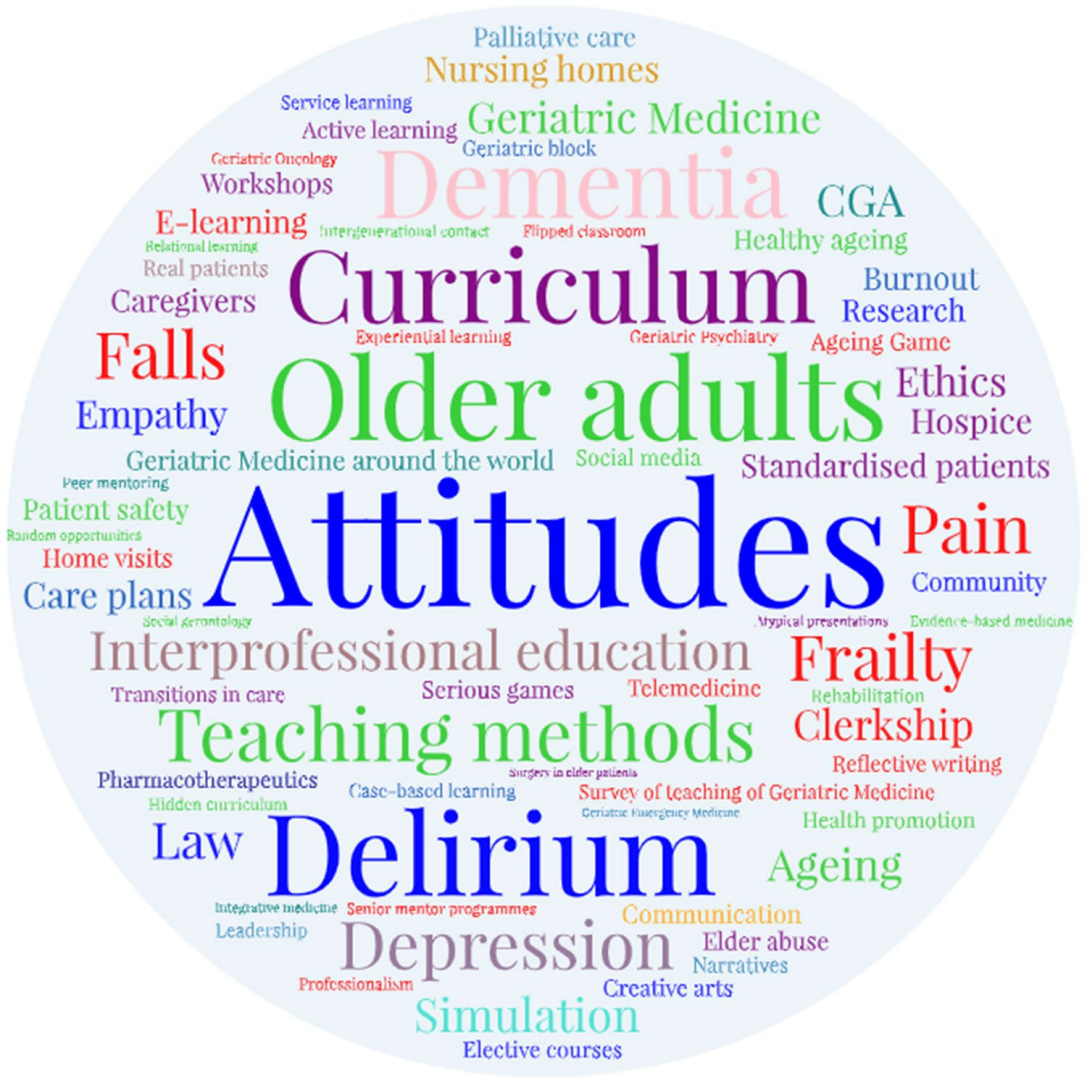
Word cloud of subthemes. This word cloud was powered by WordArt.com

We identified *six major themes* in our papers: *curricula*; *curricular topics*; *teaching methods*; *teaching settings*; medical students’ *skills*; medical students’ *attitudes* (Table 1). Figure 2 illustrates the subthemes.

**Table 1:** Classification of papers into categories of themes and subthemes

Themes	Subthemes	Table
Curriculum	Model, design Implementation General considerations Surveys	Supplementary Table 4
Topics	Caregivers Delirium, depression and dementia Elder abuse Falls and frailty Geriatric psychiatry Healthy ageing and health promotion Pain Palliative care Pharmacy Telemedicine Transitions in care Other topics	Supplementary Table 5
Teaching methods	Active learning Ageing game and serious games Case-based learning Contact with real patients Creative arts E-learning Elective courses and workshops Experiential learning Flipped classroom Geriatric block Hidden curriculum Intergenerational contact Interprofessional education Reflective learning / journaling Research Senior mentor programmes Service learning Simulation and standardised patients Other or multiple teaching methods	Supplementary Table 6
Teaching settings	Clerkship Community Long-term care settings Home visits Hospice Rehabilitation	Supplementary Table 7
Skills	Communication Empathy Leadership, moral distress and burnout, professionalism	Supplementary Table 8
Attitudes	Towards ageing, dementia and frailty Towards Geriatrics Towards older adults	Supplementary Table 9

### Curricula

Leipzig et al. developed a set of 26 Minimum Geriatrics Competencies for all graduating medical students—endorsed by the Association of American Medical Colleges (AAMC) [8]. These competencies were developed through a consensus process that involved almost half of U.S. medical schools, major medical education organisations and clinicians from several medical specialties. These competencies were nested within eight content domains: medication management; self-care capacity; falls, balance and gait disorders; hospital care; cognitive and behavioural disorders; atypical presentation of disease; health care planning and promotion; and palliative care.

Lehmann et al. built on the basic framework of the AAMC Minimum Geriatrics Competencies, by developing specific learning objectives for medical students in six geriatric mental health domains: normal ageing, mental health assessment, psychopharmacology, delirium, dementia and depression [9]. They advised vertical integration of these learning objectives in the curriculum across all years of medical school. More specifically, normal ageing and patient assessment could be covered during introductory and communication skills courses. Delirium and dementia could be included not only in geriatric or psychiatric clerkships but also in internal medicine, neurology and surgery clerkships; in this way, students would be reminded that they can encounter older adults with dementia or delirium in all clinical settings.

The Japanese core curriculum [10, 11] included a very detailed and comprehensive list of topics and competencies similar to that of Leipzig [8]. It included Comprehensive Geriatric Assessment (CGA)—a multidimensional holistic assessment of older adults—among the competencies required by newly qualified doctors; it emphasised health promotion through nutrition and falls prevention, and acknowledged the practice of the traditional Kampo medicine [11].

#### Vertical integration of Geriatric Medicine into the curriculum

Geriatric Medicine can be introduced to medical students during multiple pre-clinical and clinical courses, rather than confining it to a single Geriatric Medicine course. In this way, teaching can be reinforced through repeated exposure and core principles in the care of older people will be integrated into a wider learning. The Alpert Medical School of Brown University successfully integrated learning outcomes related to Geriatric Medicine into every course and every year for every student as part of a comprehensive curriculum redesign [12]. Principles of Geriatric Medicine were successfully incorporated into anatomy classes [13]. The Geriatrics Anatomy programme consisted of a lecture on the pathophysiology of ageing and leading causes of death and an accompanying workshop in the anatomy laboratory [13]. In the workshop, a geriatrician stimulated students to recognise common anatomical findings associated with normal ageing or disease in the anatomy cadavers of older adults (i.e. left ventricular thickening, shrinkage of the kidneys). The students positively evaluated this programme, which helped them to understand the interplay of ageing and disease.

Many *surveys* on Geriatric Medicine in undergraduate medical education have been published *worldwide* [14–17]. In 2014, a systematic review showed that the learning outcomes, academic structures and qualified teachers to support effective undergraduate teaching in Geriatric Medicine were not systematically available in most countries [14]. In Africa, a large number of medical institutions and countries did not teach Geriatric Medicine [15, 16]. In Latin America, the picture was very heterogeneous; Geriatric Medicine was taught in most undergraduate medical institutions in a few countries, while not being taught at all in other countries [17].

### Curricular topics

#### Delirium

Copeland et al. developed a curriculum for *delirium* for undergraduate medical students [18]. They detailed curricular content as well as teaching methods and settings [18]. In particular, they advised that delirium should be taught both in acute settings such as emergency departments and hospital wards, and in long-term care home settings [18].

#### Pharmacotherapeutics

Liau et al. presented educational principles related to medication management in frail older people [19]. They advocated education on frailty and “the assessment, documentation and consideration of frailty at the time that medications are prescribed, dispensed and administered”. They recommended education to minimise “low-value care”, i.e. care that provides little or no benefit to patients, has potential to cause harm, leads to unnecessary cost or unnecessary use of limited resources. In relation to medications, low-value care includes continuation of potentially futile medications associated with adverse drug events. To minimise it, they recommended appropriate deprescribing, aided by the Screening Tool of Older Persons Prescriptions in Frail adults with limited life expectancy (STOPPFrail) and Beers criteria [19]. The recent International Union of Basic and Clinical Pharmacology (IUPHAR) curriculum also included “Polypharmacy and deprescribing” among the learning topics for medical students, stating that medical students should “identify medicines to deprescribe and apply deprescribing processes in older patients” [20].

#### Healthy ageing and health promotion

Key interventions to promote *healthy ageing* include exercise prescription, management of cardiovascular risk factors and vaccination in later life. Courses on exercise prescription in older adults have been introduced in undergraduate medical education in Australia [21]. Tersmette et al. highlighted that the management of hypertension, dyslipidaemia and overweight in older adults should have different targets compared to those for younger and middle-aged adults (e.g. higher blood pressure and cholesterol targets and being mildly overweight, though not obese, may be acceptable in later life); they recommended to introduce this concept into medical curricula [22]. Education on vaccination in older age has been undertaken in France [23].

#### Frailty

According to a survey, frailty was taught in most UK medical schools, generally during geriatric ward rounds [24]. The main topics were frailty definition and diagnosis, frailty screening and assessment tools, and less frequently frailty prevention [24]. Two medical schools taught frailty through interprofessional educational workshops on prescribing [24].

*Malnutrition* in older adults has a multifactorial aetiology, is highly prevalent and associated with reduced quality of life and an increased mortality risk; yet, it can be prevented and treated through *nutritional therapy* [25]. In most European countries, doctors are responsible for prescribing nutritional therapy and referring older adults to dieticians [25]. Yet, only a few European medical schools include *nutrition in later life* in undergraduate medical curricula [25]. Teaching on causes, assessment and consequences of malnutrition has been more frequently reported than that of perioperative nutrition or nutrition in intensive care units [25].

#### Elder abuse

Kapp advocated that medical education should include legal competencies, i.e. knowledge of the law, the legal system and knowledge on how to interact with it. In particular, medical students should be aware of legal and ethical considerations relating to confidentiality, shared-decision making, surrogate authority limits and the rights of “unbefriended” patients with no one to act or advocate on their behalf [26]. These are particularly necessary when dealing with elder abuse [26]. Partnerships between academic institutions and Social Services may promote teaching of elder abuse [27].

#### Social gerontology

Tinker et al. made a call for the universal teaching on Social Gerontology in UK medical schools, asserting that this may translate to a higher quality of care for older people, as medical students become more person-centred and empathetic in approach [28]. In particular, they advocated teaching on demography, sociology of ageing, psychology of ageing and social policy. They recommended that medical students should appreciate that ageing is highly heterogeneous and influenced by economic and socio-political factors [28]. Moreover, the psychology of ageing should focus not only on diseases such as dementia but also on resilience, coping strategies and health-seeking behaviours in older age [28]. Similarly, Lehmann et al. emphasised the concepts of heterogeneity of ageing, resilience with ageing and cohort effects (i.e. those “related to the events / values / experiences of the time period during which the older patient matured”) [9]. Furthermore, Tersmette et al. highlighted the disability paradox (i.e. older adults may report well-being and social functioning despite physical and psycho-cognitive decline) [22].

#### Telemedicine

Telemedicine is a novel method of care delivery, progressively expanding across many medical specialties, in many countries. It was included in the French national curriculum [29, 30]. A compulsory module on Telemedicine was successfully implemented at the University of Zurich and shown to improve medical students’ confidence, knowledge and basic skills in Telemedicine [31]. In their feedback, most students wrote that they would like to provide telemedicine to care for chronic and older patients in their homes [31]. A French national survey showed that most medical students and residents acknowledged the relevance of Telemedicine for improving access to care but felt they were not sufficiently trained in its use [30].

### Teaching methods

*Interprofessional education* is a collaborative educational approach whereby students of two or more health or social care professions learn interactively together with the aim of providing high-quality, patient-centred care [32]. Advantages of interprofessional education for students are: understanding role delineation; expanding knowledge; learning group dynamics; feeling supported by the team; enhancing group learning; understanding other disciplines; communicating as a team [32]. Interprofessional education can take place in various settings, including residential homes, nursing homes and senior housing residences [32, 33]. For example, the Interprofessional Geriatrics Curriculum was designed to train medical students and other healthcare students to work as a team in the care of older adults in a community-based senior housing unit [33]. Collectively, by the end of it, students of all professions demonstrated a higher likelihood of understanding their roles in an interprofessional healthcare team than before it [33]. While most interprofessional learning activities emphasise knowledge and definition of roles, a few explore fluidity of roles [34].

*Senior mentor programmes* aim to promote medical students’ deeper understanding of ageing and age-associated conditions, by pairing medical students with older adults (the “mentors”). The mentors could be healthy, independent community-dwelling older adults or older adults with early dementia [35–38]. Programmes involving mentors, who represent various aspects of successful ageing, aim to broaden medical students’ concept of ageing to include healthy ageing. In the Partnering in Alzheimer’s Instruction Research Study (PAIRS) Program, medical students were paired with an adult with early-stage Alzheimer’s disease for 1 year [35]. Participation in this programme led to a modest improvement in medical students’ dementia knowledge, as shown by pre- and post-programme tests [35]. Moreover, qualitative analyses of post-programme students’ reflective essays showed that students had gained more humanistic insights into dementia, had more positive attitudes towards Geriatric Medicine and felt more comfortable with their communication skills [35]. While the PAIRS Program and many others were an elective part of the curriculum, The Time for Dementia Programme was a core element of the undergraduate medical curriculum at a UK medical school [36]. It provided all medical students with a longitudinal experience of how individuals and their families are affected by dementia, to improve students’ attitudes, compassion, empathy and knowledge of dementia [36]. In this programme, medical students visited a person with dementia and their family in pairs for 2 h every 3 months for 2 years [36].

Besides the senior mentor programmes, other programmes were reported which foster *intergenerational contact*, to support students’ understanding of ageing. For example, the Newcastle University Ageing Generations Education brought together students and older adults to discuss the subject of ageing in an educational context [39]. Following participation in this programme, students reported increased confidence communicating with older people and a better understanding of the diversity of the ageing experience [39].

*Simulation games* sensitise medical students to the challenges of older age. In the Ageing Game, students first envision themselves as ageing and older, then they are assigned various physical disabilities and play various roles to experience the world from the perspective of older adults. Finally, they have a group discussion about this [40]. The Ageing Game has been shown to improve medical students’ empathy towards older adults but to worsen attitudes [40].

*Serious games* have been used for the purposes of medical education for decades. The game GeriatriX was assessed in a controlled study at a Dutch university [41]. In GeriatriX, medical students decided which investigations and treatments to order for three older patients, all with anaemia but with different treatment preferences and levels of frailty [41]. Therefore, each patient’s optimal diagnostic and therapeutic strategy differed [41]. Students received digital feedback on their choices, based on patients’ preferences, clinical reasoning and healthcare cost [41]. After playing GeriatriX, students felt more competent in weighing patient preferences, appropriateness, and costs of medical care in complex geriatric medical decision making [41]. GeriatriX was also enjoyable and improved medical students’ attitudes toward Geriatrics [41]. Recently, *serious games* to teach delirium to medical students have been developed and tested in controlled-trials [42–44]. The serious game Delirium Experience had a positive effect on students’ skills in advising care for delirious patients, learning motivation and engagement and self-reported knowledge on delirium, while not affecting students’ attitude towards patients with delirium [44].

*Student journaling*—*reflective or narrative writing*—on teaching and clinical experiences can foster student self-reflection but also be used to assess “real-time” student attitudes and responses to curriculum redesign [12]. As an example, the Medical School of an American University integrated geriatrics into every course, every year, for every student as part of a comprehensive curriculum redesign [12]. Pre-clinical and clerkship students wrote narratives, each week or every other week, in response to standard questions on the geriatric content in medical courses, and on the older adults encountered in the various settings and during clinical experiences [12]. The narratives were analysed by a multidisciplinary team according to qualitative methods; they provided real-time feedback to course directors and allowed midcourse modifications [12]. Interestingly, a student asked to be exposed to normal ageing [12].

*Simulation* has been used to teach various topics, including delirium [45], elder abuse [46], frailty, falls and osteoporosis [47]. It has also been used to practice various skills, including communication with adults with dementia [48, 49], interprofessional team working [50], medication management [51–53], end-of-life care discussions [54], CGA [55], basic oral and dental care in older adults [56], and management of depression in older adults [57].

*Clinical placements or clerkships* are a traditional teaching and training method for medical students. Geriatric clerkships can take place in acute care hospital wards and more frequently in outpatient settings, rehabilitation units, residential and nursing homes. Geriatric clerkships can fulfil a medical school's internal medicine rotation requirement. A study found that examination proficiency was similar, while greater confidence in treating older adults and more positive attitudes towards older adults were found in the students completing a geriatric versus an internal medicine clerkship [58].

*E-learning*. A notable example of an e-learning course is Aquifer Geriatrics [59]. It consists of 26 evidence-based, peer-reviewed, yearly-updated, online case-based modules, based on the Minimum Geriatrics Competencies by Leipzig et al. [8]. The cases explore multifactorial aetiologies and multidimensional treatments. Role modelling conversations are used to teach communication skills; additional information is provided for further learning. In response to the COVID-19 pandemic many institutions restricted the availability of clinical rotation sites and deployed e-learning and distant learning to compensate for this [60, 61].

*The flipped classroom* refers to an educational model in which the teachers reverse, or “flip,” their classes so that lectures traditionally given during class are watched by students outside of class, while homework traditionally completed outside of class is completed in class [62, 63]. Specifically, students read literature and watch online lecture material prior to class, while they dedicate class time to complete activities that help deepen understanding of the lecture or apply lecture content to problem-solving. In the flipped classroom, it is the student rather than the teacher who is active during class time. The flipped classroom with online lecture material has several advantages. In particular, students may: (1) control the pace of lectures, based on their comprehension and ability levels (i.e., by re-watching lecture material that they may not fully understand while speeding through materials that they do understand); (2) re-watch lectures in preparation for exams or for personal interest and (3) not miss lectures due to illness, travel or lockdown.

*Service learning* is a form of *experiential learning*, in which students learn through tackling real-life problems in their community. Service learning can take place in various settings, including nursing homes, hospices, assisted living facilities and day centres [64–66]. In an elective service-learning course, medical students conducted needs assessments in diverse older adult communities, created health education projects and reflected on their experiences through written assignments and presentations [64]. During an arts-focussed, service-learning course, undergraduate students learnt to connect with adults with dementia in a meaningful way by interviewing them on their favourite songs and uploading them on iPods [65]. Opportunities for service-learning in nursing homes have been described also during the COVID-19 pandemic, in compliance with safety measures [66]; by restoring a sense of community, they have been mutually beneficial for both volunteer medical students and older residents in nursing homes [66].

*Short-term research training programmes* focussed on ageing have been shown to improve medical students’ attitudes toward ageing and older adults and promote interest in Geriatrics as a career [67]. In many American Universities, scholarly concentration programmes provide undergraduate medical students with opportunities for scholarship in their chosen areas of interest, beyond their traditional core curricula. These programmes allow students in-depth study, faculty mentorship and a scholarly product such as a paper or presentation. A few of these programmes have focussed on Geriatric Medicine and have been characterised by high students’ satisfaction [68].

*Creative arts*—literature, music, visual and other arts—may be used to foster communication between medical students and older adults (particularly those with dementia) [65] or to enhance students’ reflection on their thoughts and emotions sparked by their encounters with older adults [69]. In an educational intervention integrated into a mandatory clerkship, all students at Weill Medical College, Cornell University, made home visits to homebound older adults with an interdisciplinary team. Then, students channelled their thoughts and emotions elicited by the home visits into creative art projects, which were presented and discussed in interdisciplinary group discussion [69]. This group discussion allowed students to gain insight into their peers’ experiences and associated thoughts, emotions and reflections, thus promoting self-reflection and self-development [69].

### Teaching settings

*Nursing homes* are emerging as a relevant teaching setting for Geriatric Medicine [70]. Nursing homes are ideal sites for bedside teaching, interprofessional education, communication with families and education on patient safety, quality improvement projects and transitions in care (for example, between acute care hospitals and nursing homes) [70]. Copeland et al. advised that delirium teaching also should occur in long-term care homes [18]. *Home visits* allow medical students to see older adults in their own environment, thus illustrating the interplay of psychosocial and medical factors [69]. Moreover, student attitudes towards older patients may be improved by experiences that are based in the community rather than a hospital environment [69].

### Skills

Many papers focussed on communication skills, empathy and professionalism (Supplementary Table 5). Several papers focussed on communication with adults with dementia [48, 49, 65] or delirium [71] or with an end-of-life condition [72]. Liau et al. emphasised communication between care providers, patients and their caregivers in relation to medication management [19]. The development of empathy could improve medical students’ attitudes towards older adults. Visiting older adults in a nursing home fostered empathy and a more holistic approach towards older adults among medical students [73]. Medical students may experience moral distress and burnout when caring for older adults [74, 75].

### Attitudes

Several papers explored medical students’ attitudes (a) towards ageing and age-related conditions such as dementia and frailty, (b) towards older adults and (c) towards Geriatric Medicine as a career choice (Supplementary Table 4). Negative attitudes of medical students towards older adults and a low interest in Geriatrics as a career were frequently reported, with only 3–4% of students having a strong interest in Geriatrics [67, 76]. A lack of interest may be partly due to a lack of exposure to Geriatric Medicine during undergraduate education, low financial rewards and low status associated with Geriatric Medicine careers in a few countries [76]. Clerkships in Geriatric Medicine favour students’ exposure to it but it is unclear whether this results in sustained increased interest in the specialty or care of older people more generally [76]. Of note, attitudes *on caring for* older adults rather than more general attitudes *towards older adults* may better predict the choice of Geriatric Medicine as a career [77]. A medical students’ focus group highlighted perceived barriers to Geriatric Medicine as a career choice, including common ethical dilemmas, difficult communication with older patients, time-consuming assessment of older patients and what were perceived as being unrealistic expectations of patients and family members [77]. In Canada, medical students reported that the length of postgraduate training (five years) in Geriatric Medicine was a barrier to choose it as a career [76].

## Discussion

Our systematic literature search retrieved a large number of published papers on undergraduate medical education in Geriatric Medicine around the world, in the last thirteen years. We identified six major themes: curriculum; curricular topics; teaching methods; teaching settings; medical students’ skills; and medical students’ attitudes. We chose a narrative synthesis of our findings through selected papers on the main themes, due to the large number of papers, the heterogeneity of topics and the inclusion of qualitative and mixed-methods research papers, which did not allow us to perform a meta-analysis.

Model *curricula*, which were developed through a consensus process, set learning objectives and competencies for undergraduate medical students in Geriatric Medicine, Geriatric Psychiatry or specific topics [8, 9, 18].

Our review documented an *expanding teaching on topics* such as delirium, pharmacotherapeutics and deprescribing, healthy ageing and health promotion, elder abuse and legal competencies, and telemedicine. We recommend that undergraduate curricula that do not include these topics should be extended to incorporate them or adjusted to reflect the *evolving knowledge* on these.

In particular, medication management has already been included in many undergraduate curricula, including Leipzig et al. [8] and British Geriatric Society [78]. Recently, *deprescribing* has gained increasing attention. It is motivated by lack of evidence on the safety and effectiveness of drugs in older adults—remarkably underrepresented in clinical trials—and by considerations on frailty [19, 20]. Lehmann et al. stated that medical students must identify the medications that may cause or worsen cognitive impairment in older adults [9]. Liau et al. recommended education on frailty assessment in relation to medication management, especially assessing the patient’s capacity to self-manage medications, using standardised assessment tools [19]. They also recommended education to minimise low-value care, by deprescribing unnecessary or inappropriate medications and regularly reviewing medication regimens to align with changing goals of care [19]. Furthermore they specified that deprescribing should consider therapeutic intents, time to benefit, optimal dosing, adverse drug effects, risks and benefits and patient preferences [19].

Clinicians and medical students have reported limited self-efficacy in deprescribing [79], warranting further education on deprescribing and deprescribing tools. These include The American Geriatrics Society Choosing Wisely Workgroup’s publications, the updated Beers Criteria®, the FORTA (Fit fOR The Aged) List, the Screening Tool of Older People's Prescriptions and Screening Tool to Alert to Right Treatment (STOPP/START) criteria, and the Screening Tool of Older Persons Prescriptions in older adults with high fall risk (STOPPFall) [80–85]. In our view, the learning outcomes of medical students should include knowledge of *deprescribing tools* and of age- and frailty-related changing risk management and treatment goals. On the other hand, we advocate *prescription* and *optimization* of medications of proven efficacy and benefit, which could be underprescribed in older adults [86].

Furthermore, we advocate the systematic and universal teaching on *healthy ageing* and *health promotion* in medical schools. We recommend that teaching on vaccinations for older adults is included in undergraduate Geriatric Medicine courses. Currently, vaccinations are generally taught to medical students during courses of Paediatrics and Public Health, but not during Internal or Geriatric Medicine courses [87]. As a result, older adults may not appear as a target population who would benefit from effective vaccinations, including those for influenza, pneumococcus and herpes zoster [87, 88] and, now, COVID-19. In 2020, COVID-19 was the third leading cause of death in the US behind heart disease and cancer, disproportionately affecting older and frailer adults [89].

Several Authors advocated [9] and implemented [12] a *vertical integration of Geriatric Medicine into the curricula*. Given the overcrowded medical curricula, Geriatric Medicine may not only compete with other specialties for single teaching modules but also cooperate and deliver geriatric content within other specialties. This vertical integration has various motivations and advantages. First, a few topics naturally fall within and across multiple disciplines: late-life depression within Psychiatry; delirium and dementia within Internal Medicine, Neurology and Surgery; pharmacotherapeutics for older adults in Pharmacology. Second, this vertical integration would reflect the clinical reality: older adults are encountered in all clinical settings. Finally, repeated exposure to Geriatric Medicine may reinforce teaching, and exposure in a non-Geriatric Medicine setting may counteract medical students’ negative *attitudes* towards older adults. On the negative side, vertical integration may “*dilute*” Geriatric Medicine, compared with other disciplines, and its teaching may rely on non-geriatricians. Yet, co-teaching by geriatricians and non-geriatricians could be implemented, thus fostering high-quality, multidisciplinary education.

Furthermore, we advocate an *expansion of teaching settings*, beyond acute care setting. Long-term care and community settings offer great opportunities for CGA and interprofessional education and may provide medical students with a holistic view of older adults [69, 70, 73].

We have presented evidence in favour of a *variety of teaching methods* that promote learning of Geriatric Medicine. As an adjunct to traditional lectures, active and interactive learning approaches have been described. Clinical clerkships remain a cornerstone of teaching, now taking place in Geriatric Medicine acute wards, long-term care and community settings. Direct comparisons of the efficacy and effectiveness of teaching methods and settings are few [58]. Thus, we cannot recommend any one method above the others. Adopting a variety of teaching methods could maximise impact on students with different learning styles and preferences. Clinical clerkships and senior mentor programmes favour medical students’ contact with real people; simulation may ensure systematic exposure to clinical cases, rather than being determined by the older adults medical students happen to come into contact with. Moreover, the selection of teaching methods should take into account the resources available to each medical institution and the cultural context. Senior mentor programmes may not be needed in countries where intergenerational families are prevalent. We recommend a systematic curriculum redesign so that every student is exposed to Geriatric Medicine. Besides this, elective courses and workshops may be developed for students with special interest towards Geriatric Medicine.

Major strengths of our paper are the high relevance of the topic and the systematic literature search. Our comprehensive search of three electronic databases, with the support of a clinical librarian, retrieved a large number of published papers from countries across the world. No language restrictions were applied and papers in seven languages from many countries were included. We involved international experts so as not to miss other relevant published papers. Moreover, the eligible studies were selected through consensus of two or three researchers and all selected studies were theme-coded. A further strength is that we highlighted not only the contents of Geriatric Medicine that could be taught but also the variety of teaching methods used to deliver these contents and students’ attitudes that may modulate learning. Copeland et al. chose a similar approach in the development of a curriculum on delirium [18], citing that a curriculum is “a planned educational experience that encompasses behavioural goals, instructional methods and actual experiences of the learners” [90].

Our paper has a few limitations. First, we included only papers published in peer-reviewed journals, thus excluding national recommendations on learning objectives that could be published on governmental websites or medical societies’ websites [78, 91]. Second, we did not systematically carry out and present a quality appraisal of all papers; yet, we selected only papers published in peer-reviewed journals. Third, we did not systematically explore the impact of the COVID-19 pandemic on healthcare and education, though we mentioned changes in educational methods and settings in response to the pandemic [60, 61, 66]. The pandemic has favoured a shift towards Telemedicine and Telepsychiatry in clinical practice [92]; yet, few papers explored these as educational topics [29–31], without mentioning digital inequalities, which could particularly affect older adults, with cognitive or sensory impairments or simply lacking digital competencies or devices. Moreover, the pandemic has had multifold consequences on older adults, changing the prevalence and relative impact of various conditions or disease risk factors—such as loneliness, physical inactivity and barriers to access healthcare [93]. Thus, health promotion and education efforts may adjust their priorities and methods in view of the evolving pandemic.

In conclusion, efforts to promote undergraduate medical education in Geriatric Medicine have been undertaken worldwide. This review should inform future consensus-led developments or revisions of undergraduate curricula in relation to Geriatric Medicine. Further research should explore the implementation of curricula and the impact of the COVID-19 pandemic on undergraduate medical education.

## Supplementary Information

Below is the link to the electronic supplementary material.

Supplementary file1 (DOCX 91 kb)

## Data Availability

Not applicable.
