# Supplementary material for: A scoping review of the changing landscape of geriatric medicine in undergraduate medical education: curricula, topics and teaching methods
Source: Eur Geriatr Med. 2022 Jan 1;13(3):513–28. doi: 10.1007/s41999-021-00595-0 (PMC8720165; doi:10.1007/s41999-021-00595-0)
Supplement: Supplementary file 1 — Supplementary file1 (DOCX 91 kb) [file 41999_2021_595_MOESM1_ESM.docx]

**Supplementary Table 1 Search strategy used to search Ovid Medline**: **Ovid MEDLINE(R) ALL <1946 to May 17, 2021> (480 hits)**

| **Search concept** | **Search line #** | **Search strategy** | **# of results** |
| --- | --- | --- | --- |
| **Undergraduate medical students** | 1 | Education, Medical, Undergraduate/ | 24995 |
|  | 2 | education, premedical/ | 781 |
|  | 3 | Students, Medical/ | 36267 |
|  | 4 | Students, Premedical/ | 204 |
|  | 5 | (baccalaureate* adj3 (medic* or doctor)).mp. [mp=title, abstract, original title, name of substance word, subject heading word, floating sub-heading word, keyword heading word, organism supplementary concept word, protocol supplementary concept word, rare disease supplementary concept word, unique identifier, synonyms] | 41 |
|  | 6 | (bachelor* adj3 (medic* or doctor)).mp. | 252 |
|  | 7 | (undergrad* adj3 (medic* or doctor)).mp. | 29072 |
|  | 8 | (under-grad* adj3 (medic* or doctor)).mp. | 31 |
|  | 9 | (premedical adj2 student*).mp. | 354 |
|  | 10 | (pre-grad* adj3 (medic* or doctor)).mp. | 35 |
|  | 11 | (pregrad* adj3 (medic* or doctor)).mp. | 82 |
|  | 12 | (pre-medical adj2 student*).mp. | 39 |
|  | 13 | (pre-clinical adj2 student*).mp. | 287 |
|  | 14 | (preclinical adj2 student*).mp. | 761 |
|  | 15 | (student adj2 doctor*1).mp. | 182 |
|  | 16 | (medical adj2 student*).mp. | 59889 |
|  | 17 | (medical adj2 school*).mp. | 53189 |
|  | 18 | Schools, Medical | 26196 |
|  | 19 | or/1-18 | 107962 |
| **Geriatric Medicine or Ageing or Older People or Gerontology** | 20 | Geriatrics | 30470 |
|  | 21 | exp Aging/ | 252228 |
|  | 22 | geriatric*.mp. | 108291 |
|  | 23 | gerontolog*.mp. | 7859 |
|  | 24 | older people.mp. | 32357 |
|  | 25 | elder*.mp. | 284166 |
|  | 26 | seniors.mp. | 7970 |
|  | 27 | ageing.mp. | 46907 |
|  | 28 | aging.mp. | 369523 |
|  | 29 | or/20-28 | 727660 |
|  | 30 | 19 and 29 | 2131 |
| **Curriculum or Education or Learning or Training or Teaching or Competencies or Objectives** | 31 | *education/ or exp curriculum/ or education, distance/ or exp educational measurement/ or international educational exchange/ or exp teaching/ | 280848 |
|  | 32 | learning/ or problem-based learning/ or exp self-directed learning as topic/ | 89280 |
|  | 33 | "educat*".ti. | 164360 |
|  | 34 | learning.ti. | 115838 |
|  | 35 | training.ti. | 126617 |
|  | 36 | teaching.ti. | 48173 |
|  | 37 | "competenc*".ti. | 23848 |
|  | 38 | "curricul*".ti. | 17506 |
|  | 39 | objectives.ti. | 4104 |
|  | 40 | or/31-39 | 661624 |
|  | 41 | 30 and 40 | 1059 |
|  | 42 | 41 | 1059 |
|  | 43 | limit 42 to yr="2009 -Current" | 480 |
| **Total** |  |  | **480 hits** |

**Supplementary Table 2 Search strategy used to search Ovid Embase: <1974 to 2021 May 17> (1372 hits)**

| **Search concept** | **Search line #** | **Search strategy** | **# of results** |
| --- | --- | --- | --- |
| **Undergraduate medical students** | 1 | medical education/ | 225907 |
|  | 2 | bachelor* or baccalaureate* or under-grad* or undergrad* or pregrad* or pre-grad*).mp. [mp=title, abstract, heading word, drug trade name, original title, device manufacturer, drug manufacturer, device trade name, keyword, floating subheading word, candidate term word] | 65022 |
|  | 3 | 1 and 2 | 13027 |
|  | 4 | medical student/ | 73697 |
|  | 5 | premedical student/ | 95 |
|  | 6 | (baccalaureate* adj3 (medic* or doctor)).mp. | 48 |
|  | 7 | (bachelor* adj3 (medic* or doctor)).mp. | 280 |
|  | 8 | (undergrad* adj3 (medic* or doctor)).mp. | 10870 |
|  | 9 | (under-grad* adj3 (medic* or doctor)).mp. | 69 |
|  | 10 | (premedical adj2 student*).mp. | 254 |
|  | 11 | (pre-grad* adj3 (medic* or doctor)).mp. | 48 |
|  | 12 | (pregrad* adj3 (medic* or doctor)).mp. | 117 |
|  | 13 | (pre-medical adj2 student*).mp. | 51 |
|  | 14 | (pre-clinical adj2 student*).mp. | 417 |
|  | 15 | (preclinical adj2 student*).mp. | 911 |
|  | 16 | (student adj2 doctor*1).mp. | 239 |
|  | 17 | (medical adj2 student*).mp. | 89779 |
|  | 18 | (medical adj2 school*).mp. | 82381 |
|  | 19 | medical school/ | 61317 |
|  | 20 | or/3-19 | 153148 |
| **Geriatric Medicine or Ageing or Older People or Gerontology** | 21 | exp Geriatrics/ or gerontology/ | 40947 |
|  | 22 | exp Aging/ | 278198 |
|  | 23 | geriatric*.mp. | 150498 |
|  | 24 | gerontolog*.mp. | 13482 |
|  | 25 | older people.mp. | 40045 |
|  | 26 | elder*.mp. | 612525 |
|  | 27 | seniors.mp. | 10912 |
|  | 28 | ageing.mp. | 64356 |
|  | 29 | aging.mp. | 594008 |
|  | 30 | or/21-29 | 1240194 |
|  | 31 | 20 and 30 | 4436 |
| **Curriculum or Education or Learning or Training or Teaching or Competencies or Objectives** | 32 | "educat*".ti. | 174746 |
|  | 33 | learning.ti. | 130815 |
|  | 34 | training.ti. | 151072 |
|  | 35 | teaching.ti. | 53282 |
|  | 36 | "competenc*".ti. | 27691 |
|  | 37 | "curricul*".ti. | 19930 |
|  | 38 | objectives.ti. | 4245 |
|  | 39 | education/ or course content/ or curriculum/ or curriculum development/ or e-learning/ or education program/ or educational model/ or educational technology/ or educational theory/ or inclusive education/ or learning environment/ or problem based learning/ or exp simulation training/ or student retention/ or exp teaching/ or virtual learning environment/ | 598246 |
|  | 40 | learning/ or collaborative learning/ or experiential learning/ or learning curve/ or learning style/ or learning theory/ or problem based learning/ or self-directed learning/ or exp simulation training/ or skill retention/ or "transfer of learning"/ | 242143 |
|  | 41 | exp teaching/ | 96172 |
|  | 42 | 32 or 33 or 34 or 35 or 36 or 37 or 38 or 39 or 40 or 41 | 1059021 |
|  | 43 | 31 and 42 | 2139 |
|  | 44 | limit 43 to yr="2009 -Current" | 1372 |
| **Total** |  | **1372 hits** | |

**Supplementary Table 3 Search strategy used to search Pubmed: <2008 to 2021 May 17> 651 hits**

| **Search strategy** | **Automatic translations** |
| --- | --- |
| ((("doctor" or "doctors" or "medical" or "medic" or "medicine" or preclinical or premedical) and (student OR undergraduate OR pregraduate OR baccalaureate OR bachelor)) and (geriatric or gerontology or "elder" or "elders" or "seniors" or ("older people") or ageing)) and (educat*[Title] OR learn*[Title] OR curricul*[Title] OR teach*[Title]) AND (2009:2021[pdat]) | **preclinical:** "preclinical"[All Fields] OR "preclinically"[All Fields]  **student:** "student's"[All Fields] OR "students"[MeSH Terms] OR "students"[All Fields] OR "student"[All Fields] OR "students's"[All Fields]  **undergraduate:** "undergraduate"[All Fields] OR "undergraduate's"[All Fields] OR "undergraduated"[All Fields] OR "undergraduates"[All Fields]  **pregraduate:** "pregraduate"[All Fields] OR "pregraduation"[All Fields]  **baccalaureate:** "baccalaureate"[All Fields] OR "baccalaureates"[All Fields]  **bachelor:** "bachelor"[All Fields] OR "bachelor's"[All Fields] OR "bachelors"[All Fields]  **geriatric:** "geriatric"[All Fields] OR "geriatrics"[MeSH Terms] OR "geriatrics"[All Fields]  **gerontology:** "geriatrics"[MeSH Terms] OR "geriatrics"[All Fields] OR "gerontology"[All Fields] OR "gerontologie"[All Fields] OR "gerontology's"[All Fields]  **ageing:** "aging"[MeSH Terms] OR "aging"[All Fields] OR "ageing"[All Fields] |
| **Total** | **651 hits** |

**Supplementary Table 4 Papers on curricula (models, design, implementation, general considerations and surveys)**

| **Curricula, subthemes** | **Papers** | | | |
| --- | --- | --- | --- | --- |
| **Model, design** | 17 Sallehuddin, 2021  39 **Liau, 2021**  94 Sallehuddin, 2020  110 Padala, 2020  113 Naidoo, 2020  233 Mateos-Nozal, 2019  256 **Kashyap, 2019**  267 Gugliucci, 2019  290 **Anonymous, 2019***  291 Andersen, 2019  339 Meiboom, 2018 | 382 **Copeland, 2018**  398 Anonymous, 2018  401 Wilkins, 2017  448 **Lehmann, 2017**  482 Cannon, 2017  556 Gordon, 2016  607 Tohme, 2015  734 Oakley, 2014  743 **Masud, 2014**  768 Forrester-Paton, 2014  889 **Shield, 2012** | 899 Monette, 2012  900 **McNicoll, 2012**  913 Karadenizli, 2012  968 Sabzwari, 2011  999 Drootin, 2011  1009 Besdine, 2011  1010 Beer, 2011  1022 van Zuilen, 2010  1024 Tsolaki, 2010  1044 McCrystle, 2010  1048 Lee, 2010 | 1053 Just, 2010  1093 Reuben, 2009  **1110 Leipzig, 2009**  1127 Eleazer, 2009  1130 Cumbler, 2009  1133 **Blundell, 2009**  1135 Bernard, 2009  1075 **Arai, 2010** |
| **Implementation** | 260 Jaafari, 2019  979 Mathew, 2011 |  |  |  |
| **General considerations** | 295 Won, 2018  634 Michel, 2015 | 953 Wong, 2011  969 Petrovic, 2011 |  |  |
| **Surveys** | 8 Winter, 2021  17 Sallehuddin, 2021  35 Mateos-Nozal, 2021  47 Hernandez, 2021  74 **Yaghobian, 2020**  92 Shi, 2020  113 Naidoo, 2020  125 **Kotsani, 2020**  222 Nishijima, 2019 | 230 Miro, 2019  275 **Eglseer, 2019**  280 Chun, 2019  301 Vance, 2018  360 Hvalič-Touzery, 2018  426 Pati, 2017  477 Dickinson, 2017  571 Cunha, 2016  591 Amaral, 2016 | 641 Mateos-Nozal, 2015  643 **López, 2015**  673 **Frost, 2015**  742 **Mateos-Nozal, 2014**  763 Gordon, 2014  768 Forrester-Paton, 2014  799 **Tersmette, 2013**  805 Singler, 2013  859 **Dotchin, 2013** | 911 Keijsers, 2012  994 Gordon, 2011  1007 Blundell, 2011  1010 Beer, 2011  1021 Wang, 2010  1058 Gordon, 2010  1093 Reuben, 2009 |

Please, note that the number before each paper refers to the original identification number in the literature search. All these paper can be found in the Supplementary Excel file. The papers highlighted in bold are those that can be found in the References.

Anonymous, 2019 * corresponds to Reference 11.

**Supplementary Table 5 Papers on curricular topics**

| **Topics, subthemes** | **Papers** | | | |
| --- | --- | --- | --- | --- |
| **Caregivers** | 22 Rappaport, 2021  110 Padala, 2020  165 Daley, 2020 | 190 Yang, 2019  216 Pokrzywko, 2019  287 Blackie, 2019 | 293 Yanamadala, 2018  327 Pang, 2018  414 Saunders, 2017 | 492 **Banerjee, 2017** |
| **Delirium, depression and dementia** | 78 Weiss, 2020  83 Tobis, 2020  89 Stacer, 2020  110 Padala, 2020  114 Moehl, 2020  146 Gubner, 2020  165 Daley, 2020  172 **Buijs-Spanjers, 2020**  173 **Buijs-Spanjers, 2020**  210 Scott, 2019 | 214 Robles, 2019  244 Leong, 2019  331 Nimmons, 2018  360 Hvalič-Touzery, 2018  382 Copeland, 2018  389 **Buijs-Spanjers, 2018**  419 Robles, 2017  447 Lehmann, 2017  448 **Lehmann, 2017**  473 Fisher, 2017 | 492 Banerjee, 2017  511 Tinker, 2016  588 Annear, 2016  589 Annear, 2016  660 Jacinto, 2015  677 Fisher, 2015  687 Cockbain, 2015  689 Cheong, 2015  703 Alushi, 2015  796 Tullo, 2013 | 915 **Jefferson, 2012**  929 Farrell, 2012  938 Chao, 2012  958 Tullo, 2011  998 George, 2011  1010 Beer, 2011  1024 Tsolaki, 2010 |
| **Elder abuse** | 22 Rappaport, 2021 | 158 **Dyer, 2020** | 354 **Kapp, 2018** | 562 **Fisher, 2016** |
| **Falls and frailty** | 8 **Winter, 2021**  39 **Liau, 2021**  117 **McQuown, 2020**  181 Arakawa Martins, 2020 | 198 van Lierop, 2019  215 **Robles, 2019**  286 Bradley, 2019  301 Vance, 2018 | 331 Nimmons, 2018  391 Brown, 2018  430 Omlor, 2017  541 Larson, 2016 | 774 Demons, 2014  785 Atkinson, 2014  959 Sutin, 2011  1072 Boardman, 2010 |
| **Geriatric psychiatry** | 47 Hernandez, 2021  175 Blazek, 2020  216 Pokrzywko, 2019 | 280 Chun, 2019  299 Wilkins, 2018  401 Wilkins, 2017 | 447 Lehmann, 2017  448 **Lehmann, 2017**  568 De Jong, 2016 | 584 Blazek, 2016 |
| **Healthy ageing and health promotion** | 16 Shigli, 2021  21 Reilly, 2021  79 Verdeja-Vicente, 2020  136 Jadczak, 2020  143 Hash, 2020  144 Hagiwara, 2020 | 244 Leong, 2019  275 **Eglseer, 2019**  359 **Jadczak, 2018**  448 Lehmann, 2017  451 Krok-Schoen, 2017  457 Kaufman, 2017 | 459 Kaplan, 2017  466 Jadczak, 2017  518 Renschler, 2016  528 **Otsuka, 2016**  542 **Laks, 2016**  566 Diwan, 2016 | 726 Shneerson, 2014  1042 Mohler, 2010  1136 **Belmin, 2009** |
| **Pain** | 83 Tobis, 2020  114 Moehl, 2020 | 230 Miro, 2019  538 Madaus, 2016 | 714 Weiner, 2014 |  |
| **Palliative care** | 64 Bp, 2021  71 Yoshimura, 2020  192 Wilson, 2019 | 220 **Nussbaum, 2019**  365 Hayley, 2018  387 **Camp, 2018** | 471 Hall, 2017  477 Dickinson, 2017  863 Corcoran, 2013 | 982 **Lubimir, 2011**  997 Gibbins, 2011  1053 Just, 2010 |
| **Pharmacy** | 20 Reumerman, 2021  27 **Ng, 2021**  28 **Neumann-Podczaska,** **2021**  39 **Liau, 2021**  106 Poots, 2020  115 Michener, 2020 | 123 Lee, 2020  126 Kostas, 2020  164 Dallas, 2020  **256 Kashyap, 2019**  264 Hawley, 2019  448 Lehmann, 2017  541 Larson, 2016 | 569 Daughtridge, 2016  590 Anderson, 2016  656 Keijsers, 2015  706 Abd Wahab, 2015  754 Keijsers, 2014  806 Shrader, 2013  816 Ramaswamy, 2013 | 880 van Zuilen, 2012  911 Keijsers, 2012  1101 O'Connor, 2009 |
| **Telemedicine** | 7 **Wu, 2021**  28 **Neumann-Podczaska, 2021** | 57 **Ferguson, 2021**  74 **Yaghobian, 2020** | 75 **Yaghobian, 2020**  486 **Brockes, 2017** |  |
| **Transitions in care** | 19 Robertson, 2021  190 Yang, 2019 | 327 Pang, 2018  696 Bradley, 2015 | 700 Balogun, 2015  782 Block, 2014 | 1065 Eskildsen, 2010  1097 Ouchida, 2009 |
| **Other topics** | 48 Hahn, 2021  63 **Byerly, 2021**  121 Lim, 2020  143 Hash, 2020  198 van Lierop, 2019 | 222 Nishijima, 2019  272 Foguem, 2019  322 Ringer, 2018  327 Pang, 2018  354 **Kapp, 2018** | 459 Kaplan, 2017  482 Cannon, 2017  510 Turrentine, 2016  510 Turrentine, 2016  511 **Tinker, 2016** | 616 Rughwani, 2015  651 Kolb, 2015  696 Bradley, 2015  728 Salter, 2014  1022 van Zuilen, 2010 |

Please, note that the number before each paper refers to the original identification number in the literature search. All these paper can be found in the Supplementary Excel file. The papers highlighted in bold are those that can be found in the References.

Other topics include: atypical presentations of disease in older adults, care plans, comprehensive geriatric assessment, ethics and law, evidence-based medicine, Geriatric Emergency Medicine, Geriatric Oncology, integrative medicine, laws and ethics, LGBT, patient safety, social gerontology, surgery in older patients.

**Supplementary Table 6 Papers on teaching methods**

| **Teaching methods, subthemes** | **Papers** | | | |
| --- | --- | --- | --- | --- |
| **Active learning** | 100 Revell, 2020  116 **Mehta, 2020**  127 Kohn, 2020 | 144 Hagiwara, 2020  238 **Lucchetti, 2019**  244 Leong, 2019 | 314 Sauer, 2018  539 Lucchetti, 2016  580 Brand, 2016 | 960 Strano-Paul, 2011  1069 Broadfoot, 2010 |
| **Ageing game and serious games** | 172 **Buijs-Spanjers, 2020**  173 **Buijs-Spanjers, 2020**  376 Dyer, 2018 | 389 Buijs-Spanjers, 2018  415 Sauer, 2017  441 Lucchetti, 2017 | 479 de Abreu, 2017  718 **van de Pol, 2014**  751 Lagro, 2014 | 917 Huber, 2012  1077 Alfarah, 2010 |
| **Case-based learning** | 64 Bp, 2021  84 Thompson, 2020  87 Teuwen, 2020 | 115 Michener, 2020  196 van Zuilen, 2019  198 van Lierop, 2019 | 208 **Sehgal, 2019**  287 Blackie, 2019  293 Yanamadala, 2018 | 913 Karadenizli, 2012 |
| **Contact with real patients** | 396 Aquilina, 2018  697 Blanchard, 2015 | 728 Salter, 2014 |  |  |
| **Creative arts** | 93 Sevrain-Goideau, 2020  146 **Gubner, 2020** | 298 Wilson, 2018  766 George, 2014 | 881 Van Winkle, 2012  965 Shapiro, 2011 | 998 George, 2011  1047 **LoFaso, 2010** |
| **E-learning** | 19 Robertson, 2021  27 **Ng, 2021**  81 Torres, 2020  115 Michener, 2020  116 **Mehta, 2020** | 175 Blazek, 2020  447 Lehmann, 2017  538 Madaus, 2016  626 Ramaswamy, 2015  714 Weiner, 2014 | 793 Watson, 2013  816 Ramaswamy, 2013  861 Daunt, 2013  883 Tullo, 2012  938 Chao, 2012 | 1026 Tan, 2010  1061 Gillespie, 2010  1065 Eskildsen, 2010  1119 Helms, 2009 |
| **Elective courses and workshops** | 13 Sizemore, 2021  89 Stacer, 2020  298 Wilson, 2018 | 354 **Kapp, 2018**  457 Kaufman, 2017  539 Lucchetti, 2016 | 542 **Laks, 2016**  812 Rosen, 2013  979 Mathew, 2011 |  |
| **Experiential learning** | **28 Neumann-Podczaska,** 2021  71 Yoshimura, 2020  89 Stacer, 2020 | 126 Kostas, 2020  127 Kohn, 2020  158 **Dyer, 2020** | 164 Dallas, 2020  192 Wilson, 2019  590 Anderson, 2016 |  |
| **Flipped classroom** | 116 **Mehta, 2020**  368 **Granero Lucchetti, 2018** |  |  |  |
| **Geriatric block** | 427 Patel, 2017  913 Karadenizli, 2012 | 954 Visvanathan, 2011  1124 Fisher, 2009 |  |  |
| **Hidden curriculum** | 302 van de Pol, 2018  611 Shield, 2015 |  |  |  |
| **Intergenerational contact** | 7 **Wu, 2021**  54 Forster, 2021  89 Stacer, 2020 | 267 Gugliucci, 2019  414 Saunders, 2017  518 Renschler, 2016 | 566 Diwan, 2016  574 Conti, 2016  1089 Shue, 2009 |  |
| **Inter-professional education** | 13 Sizemore, 2021  16 Shigli, 2021  19 Robertson, 2021  20 Reumerman, 2021  21 Reilly, 2021  28 **Neumann-Podczaska, 2021**  42 Lee, 2021  56 Flores-Sandoval, 2021  63 **Byerly, 2021**  84 Thompson, 2020  87 Teuwen, 2020  117 **McQuown, 2020**  126 Kostas, 2020  133 Jirau-Rosaly, 2020 | 138 Holmes, 2020  151 Glassburn, 2020  158 Dyer, 2020  164 Dallas, 2020  170 Byerly, 2020  198 van Lierop, 2019  225 Nguyen, 2019  303 Truong, 2018  312 Schapmire, 2018  391 Brown, 2018  440 McManus, 2017  457 Kaufman, 2017  473 Fisher, 2017  510 Turrentine, 2016  518 Renschler, 2016 | 528 **Otsuka, 2016**  538 Madaus, 2016  539 Lucchetti, 2016  541 Larson, 2016  545 Kent, 2016  566 Diwan, 2016  574 Conti, 2016  580 Brand, 2016  588 Annear, 2016  589 Annear, 2016  590 Anderson, 2016  631 New, 2015  647 Lathia, 2015  668 Gould, 2015  700 Balogun, 2015 | 708 Yuasa, 2014  726 Shneerson, 2014  730 **Reilly, 2014**  737 Montagnini, 2014  753 Kent, 2014  764 Golden, 2014  806 Shrader, 2013  832 Lam, 2013  988 Jones, 2011  1072 Boardman, 2010  1100 Oates, 2009 |
| **Reflective learning / journaling** | 71 Yoshimura, 2020  138 **Holmes, 2020**  146 **Gubner, 2020**  173 **Buijs-Spanjers, 2020** | 192 Wilson, 2019  220 **Nussbaum, 2019**  302 van de Pol, 2018  387 **Camp, 2018** | 471 Hall, 2017  492 **Banerjee, 2017**  579 Brand, 2016  611 Shield, 2015 | 823 Nanda, 2013  863 Corcoran, 2013  889 **Shield, 2012** |
| **Research** | 77 Wilson, 2020  **357 Jeste, 2018**  699 Barron, 2015 | 939 Bragg, 2012  953 Wong, 2011 |  |  |
| **Senior mentor programmes** | 64 Bp, 2021  165 Daley, 2020 | 337 **Mendoza De La Garza,** **2018**  492 **Banerjee, 2017** | 915 **Jefferson, 2012**  947 **Basran, 2012**  1126 Eleazer, 2009 |  |
| **Service learning** | 57 **Ferguson, 2021**  146 **Gubner, 2020** | 542 **Laks, 2016**  773 Diachun, 2014 | 905 Leung, 2012 |  |
| **Simulation and standardized patients** | 15 Siew, 2021  76 **Winter, 2020**  81 Torres, 2020  117 **McQuown, 2020**  123 **Lee, 2020**  190 Yang, 2019  214 Robles, 2019 | 215 Robles, 2019  220 **Nussbaum, 2019**  227 Naylor, 2019  238 **Lucchetti, 2019**  264 Hawley, 2019  365 Hayley, 2018  391 Brown, 2018 | 419 **Robles, 2017**  528 **Otsuka, 2016**  562 **Fisher, 2016**  631 New, 2015  687 Cockbain, 2015  689 Cheong, 2015  770 Fisher, 2014 | 816 **Ramaswamy, 2013**  929 **Farrell, 2012**  959 Sutin, 2011  1002 Collins, 2011  1026 Tan, 2010  1091 Rull, 2009 |
| **Other or multiple teaching methods** | 56 Flores-Sandoval, 2021  114 Moehl, 2020  121 Lim, 2020  165 Daley, 2020  209 Sehgal, 2019  233 Mateos-Nozal, 2019  262 Holliday, 2019  286 Bradley, 2019  356 Jurivich, 2018  382 **Copeland, 2018**  389 **Buijs-Spanjers, 2018**  401 Wilkins, 2017 | 430 Omlor, 2017  441 **Lucchetti, 2017**  482 Cannon, 2017  510 Turrentine, 2016  579 Brand, 2016  616 Rughwani, 2015  647 Lathia, 2015  773 Diachun, 2014  774 Demons, 2014  779 Buhr, 2014  839 Igenbergs, 2013  856 Eckardt, 2013 | 871 Atkinson, 2013  917 Huber, 2012  968 Sabzwari, 2011  980 Mateos-Nozal, 2011  1007 Blundell, 2011  1009 Besdine, 2011  1023 Tullo, 2010  1041 Mullen, 2010  1042 Mohler, 2010  1047 **LoFaso, 2010**  1048 Lee, 2010  1087 Tung, 2009 | 1089 Shue, 2009  1093 Reuben, 2009  1097 Ouchida, 2009  1100 Oates, 2009  1101 O'Connor, 2009  1129 Denton, 2009  1130 Cumbler, 2009  1136 **Belmin, 2009**  1137 Bautista, 2009 |

Please, note that the number before each paper refers to the original identification number in the literature search. All these paper can be found in the Supplementary Excel file. The papers highlighted in bold are those that can be found in the References.

Other teaching methods include patient narratives, novice mentoring, near-peer and peer-mentoring, random opportunities, relational learning and social media.

**Supplementary Table 7 Papers on teaching settings**

| **Teaching settings, subthemes** | **Papers** | | | |
| --- | --- | --- | --- | --- |
| **Clerkship** | 216 Pokrzywko, 2019  225 Nguyen, 2019  272 Foguem, 2019  280 Chun, 2019  321 Roberts, 2018  325 Pérez-Rodríguez, 2018 | 420 Robinson, 2017  427 Patel, 2017  471 Hall, 2017  492 **Banerjee, 2017**  498 Achterberg, 2017  520 Ray-Griffith, 2016 | 569 Daughtridge, 2016  584 Blazek, 2016  588 Annear, 2016  589 Annear, 2016  902 Martinez, 2012  1060 Golden, 2010 | 1068 Diachun, 2010  1099 Olde Rikkert, 2009  1100 Oates, 2009  1115 Karasik, 2009  1116 Karasik, 2009  1137 Bautista, 2009 |
| **Community** | 198 van Lierop, 2019  244 Leong, 2019 | 542 **Laks, 2016**  566 Diwan, 2016 | 574 Conti, 2016 |  |
| **Long-term care settings** | 42 Lee, 2021  57 **Ferguson, 2021**  63 **Byerly, 2021**  71 Yoshimura, 2020  164 Dallas, 2020  170 Byerly, 2020  192 Wilson, 2019  225 Nguyen, 2019 | 238 **Lucchetti, 2019**  414 Saunders, 2017  420 Robinson, 2017  545 Kent, 2016  565 **Edirne, 2016**  584 Blazek, 2016  588 Annear, 2016  589 Annear, 2016 | 606 Tong, 2015  662 Huls, 2015  728 Salter, 2014  779 Buhr, 2014  836 Kalender-Rich, 2013  841 Helmich, 2013  847 Gugliucci, 2013  848 Gillespie, 2013 | 893 Roane, 2012  902 Martinez, 2012  **914 Kanter, 2012**  1005 Buhr, 2011  1129 Denton, 2009 |
| **Home visits** | 21 Reilly, 2021  158 **Dyer, 2020** | 518 Renschler, 2016  574 Conti, 2016 | 1079 Abbey, 2010  1087 Tung, 2009 |  |
| **Hospice** | 57 **Ferguson, 2021** | 863 Corcoran, 2013 |  |  |
| **Rehabilitation** | 1137 Bautista, 2009 |  |  |  |

Please, note that the number before each paper refers to the original identification number in the literature search. All these paper can be found in the Supplementary Excel file. The papers highlighted in bold are those that can be found in the References.

**Supplementary Table 8 Papers on medical students’ skills**

| **Skills, subthemes** | **Papers** |
| --- | --- |
| **Communication** | 39 **Liau, 2021**  54 **Forster, 2021**  76 **Winter, 2020**  89 Stacer, 2020  93 Sevrain-Goideau, 2020  110 Padala, 2020  146 **Gubner, 2020**  414 Saunders, 2017  563 **Fisher, 2016**  569 Daughtridge, 2016  687 **Cockbain, 2015**  982 **Lubimir, 2011**  1002 Collins, 2011 |
| **Empathy** | 368 **Granero Lucchetti, 2018**  376 Dyer, 2018  387 **Camp, 2018**  441 **Lucchetti, 2017**  415 Sauer, 2017  565 **Edirne, 2016** |
| **Leadership, moral distress and burnout, professionalism** | 79 Verdeja-Vicente, 2020  108 **Perni, 2020**  387 **Camp, 2018**  611 Shield, 2015 |

Please, note that the number before each paper refers to the original identification number in the literature search. All these paper can be found in the Supplementary Excel file. The papers highlighted in bold are those that can be found in the References.

**Supplementary Table 9 Papers on medical students’ attitudes**

| **Attitudes, subthemes** | **n** | **Papers** | | | |
| --- | --- | --- | --- | --- | --- |
| **Towards ageing, dementia and frailty** | **15** | 34 McCarthy, 2021  42 Lee, 2021  45 Jester, 2021  134 Jester, 2020 | 210 Scott, 2019  336 Merz, 2018  467 Jacinto, 2017  479 de Abreu, 2017 | 584 Blazek, 2016  672 Fujii, 2015  703 Alushi, 2015  766 George, 2014 | 812 Rosen, 2013  915 **Jefferson, 2012**  998 George, 2011 |
| **Towards Geriatrics** | **35** | 42 Lee, 2021  45 Jester, 2021  47 Hernandez, 2021  92 Shi, 2020  111 Nuss, 2020  142 Hebditch, 2020  144 Hagiwara, 2020  151 Glassburn, 2020  216 Pokrzywko, 2019 | 302 van de Pol, 2018  325 Pérez-Rodríguez, 2018  338 Meiboom, 2018  345 Lucchetti, 2018  440 McManus, 2017  520 Ray-Griffith, 2016  565 Edirne, 2016  579 Brand, 2016  606 Tong, 2015 | 636 Meiboom, 2015  **637 Meiboom, 2015**  699 Barron, 2015  724 Soethout, 2014  781 Boyle, 2014  812 Rosen, 2013  821 Ni Chroinin, 2013  854 Farrell, 2013  858 Duque, 2013 | 869 Bensadon, 2013  915 **Jefferson, 2012**  935 Decherrie, 2012  962 Singh, 2011  988 Jones, 2011  1016 Zwahlen, 2010  **1074 Bagri, 2010**  1084 Weiss, 2009 |
| **Towards older adults** | **55** | 34 McCarthy, 2021  151 Glassburn, 2020  296 Wilson, 2018  297 Wilson, 2018  302 van de Pol, 2018  319 Ross, 2018  336 Merz, 2018  337 **Mendoza De La Garza, 2018**  357 Jeste, 2018  368 **Granero Lucchetti, 2018**  383 Chepo Chepo, 2018  415 Sauer, 2017  440 McManus, 2017  441 **Lucchetti, 2017** | 574 Conti, 2016  580 Brand, 2016  615 Ruiz, 2015  636 Meiboom, 2015  637 **Meiboom, 2015**  652 Koh, 2015  712 Wiese, 2014  718 **van de Pol, 2014**  720 Tam, 2014  730 **Reilly, 2014**  764 Golden, 2014  765 Goeldlin, 2014  819 Ozcan, 2013  822 **Nguyen, 2013**  854 Farrell, 2013 | 858 Duque, 2013  871 Atkinson, 2013  881 Van Winkle, 2012  888 Stall, 2012  889 Shield, 2012  893 Roane, 2012  905 Leung, 2012  908 Kumar, 2012  909 Koh, 2012  921 Higashi, 2012  947 Basran, 2012  965 Shapiro, 2011  979 Mathew, 2011  1023 Tullo, 2010  1029 Snyder, 2010 | 1041 Mullen, 2010  1059 Gonzales, 2010  1060 Golden, 2010  1068 Diachun, 2010  1077 Alfarah, 2010  1079 Abbey, 2010  1089 Shue, 2009  1112 Klaghofer, 2009  1125 Eskildsen, 2009  1126 Eleazer, 2009  1131 Cheong, 2009 |

Please, note that the number before each paper refers to the original identification number in the literature search. All these paper can be found in the Supplementary Excel file. The papers highlighted in bold are those that can be found in the References.
